# Efficiency and Productivity Differences in Healthcare Systems: The Case of the European Union

**DOI:** 10.3390/ijerph20010178

**Published:** 2022-12-22

**Authors:** Roman Lacko, Zuzana Hajduová, Tomáš Bakalár, Henrieta Pavolová

**Affiliations:** 1 Department of Tourism, Faculty of Commerce, University of Economics in Bratislava, Dolnozemská Cesta 1, 852 35 Bratislava, Slovakia; 2 Department of Business Finance, Faculty of Business Management, University of Economics in Bratislava, Dolnozemská Cesta 1, 852 35 Bratislava, Slovakia; 3 Faculty of Mining, Ecology, Process Control and Geotechnologies, Technical University of Košice, Letná 9, 042 00 Košice, Slovakia

**Keywords:** MPI, DEA, clustering, healthcare, efficiency

## Abstract

This study aims to identify significant differences between the countries of the European Union, follow the course of achievement of the convergence objectives, assess developments against specific common characteristics of the countries, and propose possible measures that could improve the state of health in the EU as a whole by implementing standard cohesion policies. To compare efficiency and productivity among the states of the European Union, we used data envelopment analysis (DEA) and the Malmquist productivity index (MPI). On the basis of our findings, even countries that joined the EU later achieve high technical efficiency values. However, it should be noted that it is in these countries that technical efficiency values tend to decline. The values of the Malmquist productivity index broadly indicate stagnation in western countries and productivity decline in central and eastern European countries. This decline is mainly due to a negative shift in the technological frontier in these countries.

## 1. Introduction

The efficiency of healthcare systems and the use of financial and other resources are very often discussed in not only the scientific but also the practical and political spheres. This is also indicated by literature surveys regularly published in various journals. Therefore, measuring efficiency in healthcare is linked to the search for variables that can plausibly reflect the level and quality of healthcare in countries, regions, or health service providers.

Since health is one of the most crucial sectors of the public sector, drawing the attention of national governments and political groups to improve their health is an inevitable phenomenon. Healthcare has also found a place in sustainable development goals and recovery plans. Today, people are even more aware of the importance of this sector, the imperfections of which have been revealed by the COVID-19 pandemic [1].

### 1.1. Healthcare in the European Union

Research suggests that inequalities between countries are significant and see sustainable economic growth as one of the options for improving health [2]. Healthcare is closely linked to different spheres of national economies. Scientists have even demonstrated the connection between economic crises and the growth of amenable deaths. Research also indicates differences between European countries, with Eastern European countries lagging far behind in the quality of healthcare provision [3]. This inequality is also reflected in the efficiency of countries, but it must be stressed that even countries whose healthcare is perceived at a high level may not be efficient [4]. As healthcare is increasingly pushing to reduce costs and improve healthcare, which highlights the importance of efficiency, these effects are more pronounced during crises. However, efficiency gains will often occur at the expense of the quality of healthcare [5].

For this reason, financial stability and sustainability are often mentioned, given many factors—population aging, rising investment prices, and pressure on salary growth [6,7]. Thus, we often encounter views that call for thorough research on the efficiency of spending resources on healthcare, such as the value-for-money approach and cost–benefit analysis [8]. This research concerns the efficiency of the European Union (EU) countries. In addition, research is investigating facts and contexts related to healthcare spending in EU countries [9,10,11]. This area and research subject are also of interest because they are public resources, and countries should also pursue convergence objectives together with the institutions of the European Union [9].

### 1.2. Efficiency Measurement in Healthcare

As we have already indicated above, healthcare efficiency is a discussed topic, and there are several approaches to measuring efficiency. Efficiency can be measured on the part of healthcare providers [12,13,14], countries, and regions [15,16], as well as political groupings [17,18,19,20]. Many studies use the Malmquist productivity index (MPI) to reflect the change in healthcare systems [21]. Both DEA and MPI can contribute to the selection of proper policy measures in individual states and groupings [22,23,24,25]. In our study, we address the political grouping of the European Union, where many types of research have already been carried out. Some studies proposed a decline in technical efficiency and productivity in selected regions [18]. Since the measurement is a continuous process in a dynamic environment of healthcare, it must be performed regularly, and a comprehensive evaluation of the EU healthcare systems is missing.

In this study, addressing the research gaps identified above, we analyze developments, changes, and reasons for efficiency changes with productivity in selected European countries during the period under review. In addition, this study looks for significant differences between the countries of the European Union, follows the direction toward the convergence objectives, assesses developments against specific common country characteristics, and proposes possible measures that could improve the state of health in the EU as a whole by implementing standard cohesion policies.

This study is divided into five sections. In Section 1, we discuss the current state of the art in the field of measuring efficiency in healthcare and healthcare problems in general. In Section 2, we described methods used to measure efficiency and the object of the research. A concise review of variables and the data is also presented in Section 2. In Section 3, we present the results of the DEA and MPI models. Moreover, cluster analysis of the individual efficiency results is presented. In Section 4, the reasons and possibilities for the results differences are discussed. In Section 5, a conclusion, policy implications, and future research options are proposed.

## 2. Materials and Methods

### 2.1. Data Envelopment Analysis and Malmquist Productivity Index

On the basis of research and other literature surveys [26,27,28,29], the most commonly used method for evaluating efficiency in healthcare is the method of data envelopment analysis along with its modifications. The Malmquist productivity index is often used to assess productivity and change over time based on the DEA method.

Its theoretical foundations were laid by Farrell [30] and developed in many other studies. Further studies have contributed to significant theoretical development [31,32,33]. They proposed models that assume either constant returns on the scale—CCR (CRS) DEA models (1) or models that assume variable returns on the scale—BCC (VRS) DEA models (2). We use both models in this study. The main difference is in the shape of the efficiency frontier; in the CRS model, the frontier is a linear line, whereas, in the VRS model, it has a nonlinear shape. In this study, we only use DEA models focused on inputs because they are more likely to be changed by policymakers. For the decision-making unit (DMU) to be efficient, it must achieve efficiency equal to 1.

(1) $$\begin{array}{c} \underset{\theta_{B},\;\lambda }{\min}\theta_{B}\\ s.t.{\;\theta }_{B}x_{o}-X\lambda \geq 0\\ Y\lambda \geq y_{o}\\ \lambda \geq 0, \end{array}$$

(2) $$\begin{array}{c} \underset{\theta_{B},\;\lambda }{\min}\theta_{B}\\ s.t.{\;\theta }_{B}x_{o}-X\lambda \geq 0\\ Y\lambda \geq y_{o}\\ e\lambda =1\\ \lambda \geq 0, \end{array}$$

where $\theta_{B}$ is a real efficiency value, $X=\left(x_{j}\right)\in R^{m\times n}$ and Y = $\left(y_{j}\right)\in R^{s\times n}$ are a given set of data, e is a row vector in which all elements are equal to 1, $\lambda \in R^{n}$ is a non-negative vector, and $x_{o}$ and $y_{o}$ are positive input and output vectors.

The Malmquist productivity index (MPI) measures productivity changes along with time changes and can be broken down into efficiency changes and changes in technology using a DEA nonparametric approach. MPI can be expressed using the distance function (E) as Equations (3) and (4) using observations at time *t* and *t* + 1.

(3) $$MPI_{I}^{t}=\frac{E_{I}^{t}\left(x^{t+1},y^{t+1}\right)}{E_{I}^{t}\left(x^{t},y^{t}\right)},$$

(4) $$MPI_{I}^{t+1}=\frac{E_{I}^{t+1}\left(x^{t+1},y^{t+1}\right)}{E_{I}^{t+1}\left(x^{t},y^{t}\right)},$$

where *x* is an input vector, *y* is an output vector, and *I* indicates the model orientation (input). The geometric mean of the MPI from Equations (3) and (4) can then be calculated as shown in Equation (5).

(5) $$MPI_{I}^{G}={\left(MPI_{I}^{t}\times MPI_{I}^{t+1}\right)}^{1/2}={\left[\left(\frac{E_{I}^{t}\left(x^{t+1},y^{t+1}\right)}{E_{I}^{t}\left(x^{t},y^{t}\right)}\right)\times \left(\frac{E_{I}^{t+1}\left(x^{t+1},y^{t+1}\right)}{E_{I}^{t+1}\left(x^{t},y^{t}\right)}\right)\right]}^{1/2}.$$

This geometric mean can then be divided into so-called “technological change” (TECH)—change in technological efficiency (TE) and change in efficiency (EFFCH)—change in management efficiency (ME); see Equation (6).

(6) $$MPI_{I}^{G}={\left(EFFCH_{I}\times TECHCH_{I}^{G}\right)}^{1/2}=\left(\frac{E_{I}^{t+1}\left(x^{t+1},y^{t+1}\right)}{E_{I}^{t}\left(x^{t},y^{t}\right)}\right)\times {\left[\left(\frac{E_{I}^{t}\left(x^{t},y^{t}\right)}{E_{I}^{t+1}\left(x^{t},y^{t}\right)}\right)\times \left(\frac{E_{I}^{t}\left(x^{t+1},y^{t+1}\right)}{E_{I}^{t+1}\left(x^{t+1},y^{t+1}\right)}\right)\right]}^{1/2}.$$

Technological change is due to technological changes (investments in new machinery and buildings). Management decisions cause a change in efficiency. If the indicator value is greater than 1, it means that there has been an efficiency gain; if the value is less than 1, it means that there has been a decrease in efficiency [34].

### 2.2. Research Object and Data

The object examined in this study constituted the countries of the European Union. Today, the European Union consists of 27 countries. In this research, we omitted Malta, Cyprus, and Luxembourg because of the unavailability of data. We examined the remaining 24 countries for the period 2013–2019. This is how we created a data panel with a size of 216 observations—decision-making units (DMU).

We obtained data for our research from the databases of the European Statistical Office [35], as they contained the most consistent data sources. As input variables, we selected two variables on the basis of previous studies. The first variable represents capital resources—healthcare expenditures (HC_Exp) [11,19,36] for long-term healthcare denominated in purchasing power standard per capita. The second variable represents capacity resources—number of hospital beds (Hosp_Beds) relative to 100,000 inhabitants. [4,17,18]. These two inputs represent two major production factors—capacity and capital. Countries which spend fewer resources and/or deal with lower hospital capacity and produce the same number of outputs or even higher must be more efficient than those with higher amounts of inputs. In the research, we originally planned to use an input characterizing the number of people working in medical fields; however, because of the unavailability/inconsistency of data, we decided not to use this input. In addition, such an entry would have significantly reduced the number of countries surveyed and years. Table 1 shows selected statistical indicators of the inputs used for each country.

The highest average spending in the reporting period was in Germany, The Netherlands, and Sweden, along with others mostly in western and northern Europe. By contrast, the lowest expenditure expressed in PPS per capita was in Romania, Bulgaria, and Latvia. Differences in minimum and maximum levels were not significant, but it must be noted that most countries’ spending on the country’s health systems has increased.

Germany, Austria, and Bulgaria had the highest number of hospital beds per 100,000 inhabitants. The lowest average bed counts were in Denmark, Sweden, and Spain. Although a high number of beds does not necessarily mean quality healthcare, the number of beds should be reduced to such levels that healthcare is provided for all who need it. In most countries, there has been a significant decrease in the number of beds.

We consider three variables as outputs in this research. We can consider the number of outpatients and inpatients as output. A higher number of treated persons must not indicate healthcare efficiency; it must also be due to low prophylaxis levels. There are indicators which reflect the quality of the healthcare system according to the health status of its inhabitants. The first variable is the number of years in full health at age 65 (Health_LY_65); it measures the number of years that a person at age 65 is still expected to live in a healthy condition. It is a health expectancy indicator which combines information on mortality and morbidity. This variable indicates the state of the health system in earlier periods. It is an output that faithfully indicates the previous development of the health system’s quality. It also indicates the state of health of the country’s population. The second and third output variables are the life expectancy of men (Life_Expect_M) and women (Life_Expect_F) at birth [17,18,19,37] in absolute numbers, i.e., how long is a person expected to live after birth. The variable had to be broken down by gender as overall averages were unavailable. This variable plausibly displays the quality of crushing systems at present. Table 2 shows selected statistical indicators of output variables per country.

The highest number of healthy years at the age of 65 was recorded in Sweden, Ireland, and Denmark. The lowest number of years in full health at the age of 65 was recorded is Latvia, Slovakia, and Croatia. Life expectancy at birth was the longest for both men and women in Spain, Italy, and Sweden. The lowest values were recorded in Bulgaria, Romania, and Hungary. For all three output variables, an increase in absolute values can be observed over the period under review, which is a positive fact. Individual measurements were processed through the R program.

## 3. Results

**Figure 1:**
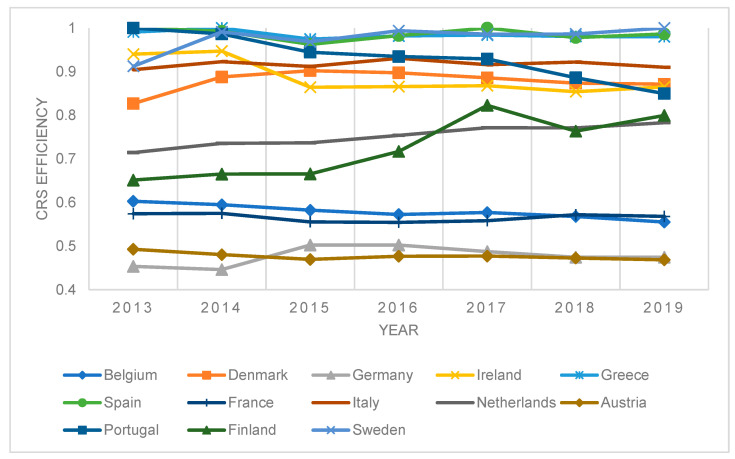
Results of DEA window efficiency measurement—CRS model, selected countries.

In this section, we characterize the results of efficiency modeling using the DEA method and productivity changes using MPI. For the needs of this research and clarity of results and context, we divide the results of the technical efficiency modeling into countries that joined the EU before and after 2014 inclusive. Efficiency was measured for countries together, where we took the window approach. Figure 1 describes the results of efficiency modeling for the input model assuming CRS and countries that joined the EU before 2004.

**Figure 2:**
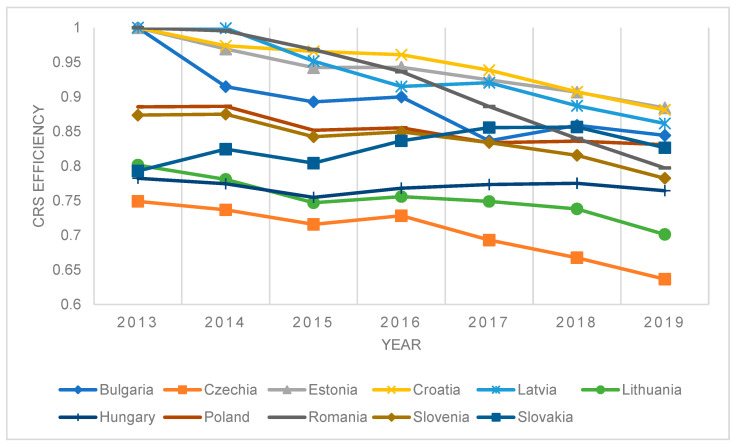
Results of DEA window efficiency measurement—CRS model, selected countries.

The efficiency of using capacity and financial resources, taking into account the quality of the health of the country’s population, has been largely stagnant in these countries. The least efficient countries were Austria and Germany, which spent relatively high resources on healthcare. On the other hand, Greece, Spain, and Sweden were among the most efficient countries, with the most efficient being Finland and The Netherlands. By contrast, the most significant decline in efficiency was observed in Portugal. Figure 2 describes the results of efficiency modelling for the input model assuming CRS and countries that joined the EU after 2004 inclusive.

The situation was slightly different in the countries that joined the EU at a later stage, where most countries had declined technical efficiency. This suggests that extensive spending was not translated into the quality of the population’s health quickly enough or sufficiently efficiently in these countries. Croatia, Estonia, and Latvia achieved the highest efficiencies. The Czech Republic and Lithuania were among the least efficient countries. However, they were more efficient than the weakest countries that joined the EU before 2004. In general, it can be concluded that the efficiency of the countries that joined the EU later was not so significant compared to the original EU countries. However, there is a significant difference in efficiency decline.

**Figure 3:**
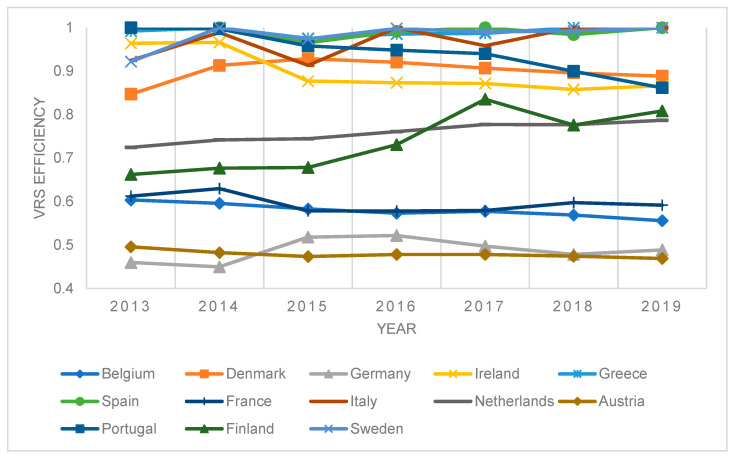
Results of DEA window efficiency measurement—VRS model, selected countries.

Figure 3 shows the results of efficiency modeling for the input model, assuming the VRS and the countries that joined the EU by 2004.

**Figure 4:**
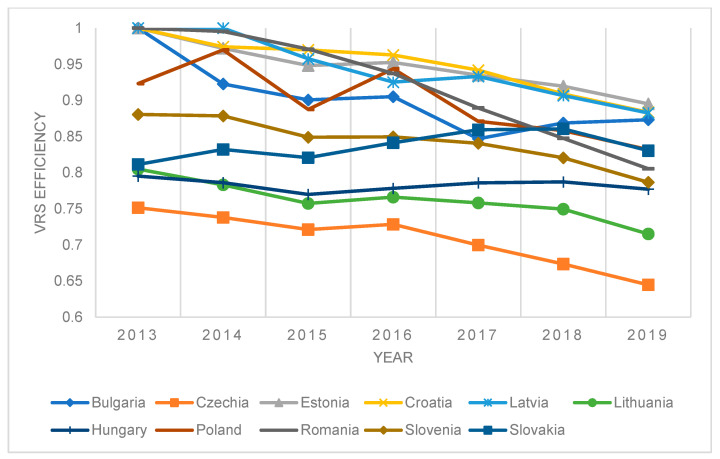
Results of DEA WINDOW efficiency measurement—VRS model, selected countries.

Figure 4 shows the results of efficiency modeling for the input model assuming VRS and countries that joined the EU after and within 2004.

Table 3 summarizes the country-specific DEA results of the CRS and VRS models. Above the line are the countries that joined the EU after 2004 inclusive.

In this summary table, it can be observed that the efficiency values of the countries that joined the EU in recent years were on average (0.8525) higher than the averages of countries that joined the EU before 2004 (0.7799). Estonia, Croatia, Sweden, Spain, Greece, Estonia, Croatia, Sweden, Spain, and Greece had the highest average efficiency values over the reporting period, but other countries were not far behind.

Standard deviations indicated low variability in technical efficiency values for most countries surveyed. Table 4(A,B) provides the result of the MPI measurement of productivity change over time for the whole period under review.

Many facts can be established on the basis of the presented results of measuring the changes in productivity in EU countries over time. First, productivity growth over the whole period was observed only in Sweden. On the contrary, some countries, such as Estonia and Romania, experienced a steady decline in productivity. For countries that joined the EU before 2004, the geometric mean change in productivity (MPI) was higher throughout the reporting period. Another interesting fact is the trend of higher values of geometric mean changes caused by technology efficiency change (TechCh) in countries that joined the EU later. Conversely, countries that joined the EU before 2004 had higher average changes in efficiency caused by changes in technical efficiency (EffCh). In general, in addition to 2015/2016, there was a decline in EU countries’ productivity in financing health systems during the reporting period.

**Figure 5:**
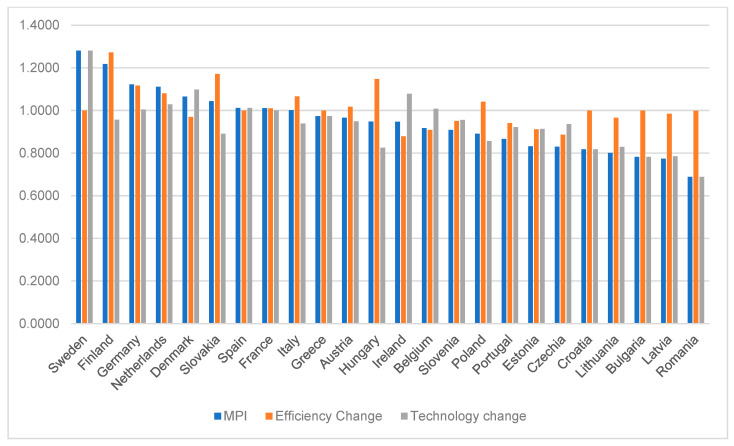
Changes in productivity between the years 2019 and 2013.

To better understand the context, we examined productivity changes directly between 2019 and 2013, i.e., we omitted 2014–2018 in the calculations. The resulting MPI values, technical efficiency changes, and changes in technology efficiency are shown in Figure 5.

**Figure 6:**
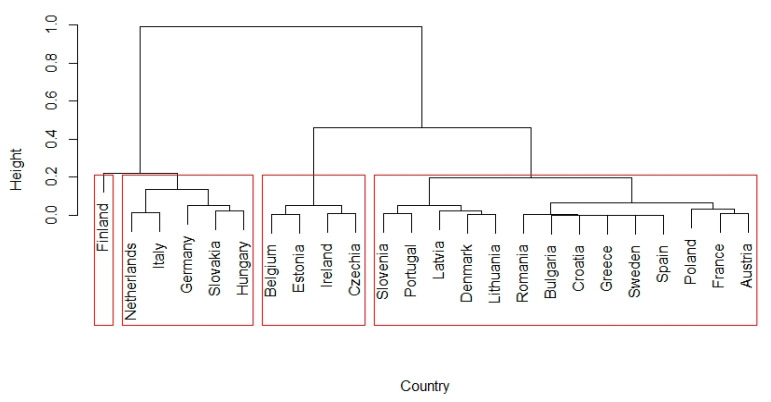
Cluster dendrogram of technical efficiency change (EffCh) between the years 2019 and 2013.

MPI values were used to arrange countries in descending order. The highest increases in MPI were recorded in Sweden (1.2805), Finland (1.2177), and Germany (1.1227). The most significant decreases in productivity were recorded in Romania (0.6883), Latvia (0.7738), and Bulgaria (0.7823). The turning point in the increase/decrease in productivity was between Italy and Greece, where MPI values decreased. Our previous findings are corroborated by the fact that the decline in productivity in countries that joined the EU after 2004 was mainly driven by a significant reduction in technology efficiency (TechCh). In countries such as Slovakia, Hungary, and Poland, the decline in the efficiency of technology was limited by the increase in technical efficiency. To further investigate the effects of TechCh and EffCh, we decided to push up clusters of countries and look for common causes of this situation. Figure 6 shows the cluster analysis results for the change in technical efficiency between the beginning and the end of the period under review (EffCh).

**Figure 7:**
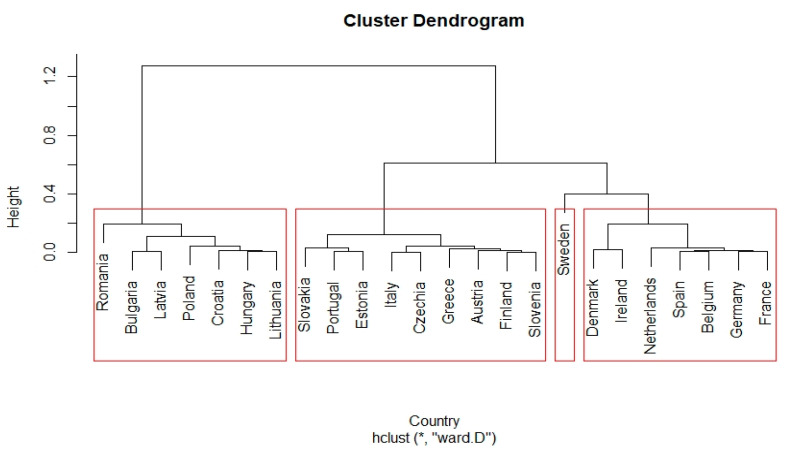
Cluster dendrogram of change of the efficiency of technology (TechCh) between years 2019 and 2013.

Using the so-called elbow method, we identified the possibility to create four clusters with the countries we surveyed. A separate cluster consisted of Finland, which achieved the highest productivity gains due to the increase in brick efficiency. This was followed by a cluster of five countries belonging to the original EU countries and those that joined the EU in 2004. There were also four countries in the third cluster. Lastly, the fourth cluster, where up to 14 countries can be found, was extensive. In these countries, the change in EffCh was around 1.0. In most cases, productivity dropped due to decreased technical efficiency. Figure 7 shows the cluster analysis results for changing the technology efficiency between the beginning and the end of the period under review (TechCh).

Using the so-called elbow method, we identified the possibility to create four clusters with the countries we surveyed. The first cluster consisted of Sweden, while the second cluster of countries with lower technology efficiency changes consisted of seven countries. It should be noted that some of them achieved a negative change, i.e., a decrease in TechCh values. It is in this cluster that only indigenous EU countries were still represented. Another cluster comprised a mix of countries that joined the EU before and after 2004. Only countries that joined the EU in 2004 or later were in the last cluster. The countries of this cluster experienced the highest decrease in TechCh.

## 4. Discussion

Both previous research and our research have confirmed that cohesion policy’s convergence objectives in healthcare do not fulfill their potential to a sufficient degree [10]. We take a very positive view that in most countries the values of technical efficiencies are at an excellent level, but the problem is the decline in technical efficiency, mainly in the countries of the “eastern” bloc. Our research also confirmed other research that suggests an increase in technical efficiency but a negative shift in a technological frontier [5]. In our case, the shift of the technological frontier was further decomposed, and we found that the leading cause was the countries of central and eastern Europe. This effect may be because resources invested in various facilities, buildings, and other capital expenditures are not invested efficiently. This can be due to excessive waste of resources, lack of value for money, corruption, and many other problems.

On the contrary, technical efficiency did not change significantly in the countries surveyed and showed a growing trend rather than declining. This can be due to the excellent quality of the services provided by the financial sources. We are, therefore, faced with a significant problem, particularly evident in the countries that joined the EU later. The problem is that we have quality pearls, but we do not have or do not use capital efficiently enough for capital investments.

Countries such as Germany and Austria, which achieved relatively low efficiencies, cannot be considered countries with poor healthcare quality. In their case, inputs are only transferred to a lesser extent to the outputs. Our research did not include, for example, patient satisfaction and the quality of healthcare facilities, as these indicators are relatively difficult to measure and unavailable for all countries on a unified basis.

Efficiencies in countries that joined the EU in 2004 may also be due to the outflow of medical staff in better conditions abroad. Some countries are still unable to compete with the more developed countries, especially regarding salaries and working conditions. However, as other authors recommended, these factors need to be analyzed at the national level [20], because, even at the national level, there may be a sizeable interregional inequality [38]. We must also think that healthcare providers are the primary source of inefficiencies; therefore, better management of health service providers needs to be addressed [39]. Lastly, we must not forget about the heterogeneity of health systems [9]. Even according to our research, it seems that systems, where part of the reimbursement is on the patient, are more efficient. This system motivates citizens to better prophylaxis.

## 5. Conclusions

On the basis of the results and discussion, many conclusions can be drawn, which may also be recommendations for policymakers.

As for efficiency in the countries that joined the EU in 2004 and later declined, it is necessary to rethink cohesion policy and the direction of inventiveness from the structural funds to those countries. These countries are still actual beneficiaries of structural funds resources. Investments should be aimed at maintaining a high-quality workforce in those countries, which firmly holds the efficiency of these countries at acceptable levels. Encourage investment in buildings, infrastructure, and equipment, as these countries are still lagging behind western European countries in this area. These efforts can also be seen in the current recovery plans, where some countries want to have completely new hospitals and medical facilities through structured funds.

Furthermore, the various post-pandemic recovery support plans need to be geared toward specific regions. Our research has also shown still significant differences in countries, which may also result from different ways health systems are financed even in last years, not only in the years we researched. Countries should not be allowed to use relatively costly resources in an address manner.

This study dealt with some limitations. The first and most relevant was the absence of human capital input. Many studies use the labor input (such as medical staff) but we were unable to find consistent data for this indicator on the EU level. We can only exclude some countries or periods, which would, in our opinion, lead to inconsistent conclusions on the EU policies level. Many countries must deal with low numbers of medical staff; hence, excluding this indicator as input did not excessively harm the results.

It can be observed that health spending in countries grew relatively sharply every year. This guides our recommendation to improve the quality of datasets, which are often incomplete and sometimes lack countrywide data. This prevents a more detailed analysis of the causes of the inequalities that have arisen. Similarly, we could analyze how efficiently they are handled with the structural funds. Future research could also include the measurement of scale changes in both CRS and VRS models using individual clusters, which could help describe the differences more thoroughly. This could help both the scientific sphere and the makers of standard cohesion policies.

## Figures and Tables

**Table 1:** Descriptive statistics of the inputs.

Country	HC_Exp			Hosp_Beds		
	Units: PPS per Capita			Units: Per 100,000 Capita		
	Mean	Min	Max	Mean	Min	Max
Belgium	3661.37	3350.55	3901.29	574.69	556.72	592.76
Bulgaria	1161.39	936.19	1316.56	731.64	681.64	774.07
Czechia	2079.55	1849.43	2442.58	664.79	658.04	670.16
Denmark	3635.87	3365.53	3914.99	267.12	253.01	307.06
Germany	4208.83	3739.93	4658.60	808.60	791.48	827.77
Estonia	1515.09	1235.78	1791.88	471.10	453.01	490.29
Ireland	3382.48	3199.83	3633.14	283.40	256.00	297.39
Greece	1612.05	1537.57	1657.44	421.62	418.01	424.60
Spain	2341.29	2087.76	2573.23	296.67	294.60	297.92
France	3615.22	3436.42	3769.67	605.55	583.79	627.18
Croatia	1234.41	1040.18	1439.61	566.27	549.25	590.62
Italy	2431.03	2298.40	2611.20	319.63	314.05	331.17
Latvia	1172.09	928.81	1457.48	562.21	542.32	579.98
Lithuania	1564.48	1255.78	1949.20	679.60	634.65	731.25
Hungary	1464.64	1375.88	1550.80	698.49	690.75	703.73
Netherlands	3850.99	3719.12	4101.99	338.69	307.84	369.25
Austria	3841.69	3600.69	4077.62	743.05	718.90	764.46
Poland	1434.59	1261.98	1636.24	654.94	617.45	664.04
Portugal	2101.99	1891.45	2392.51	340.23	331.91	350.60
Romania	988.81	781.79	1354.42	684.77	667.31	705.75
Slovenia	2056.00	1850.30	2361.10	449.26	442.79	455.20
Slovakia	1533.19	1434.00	1626.74	577.11	569.62	582.05
Finland	3082.16	2970.87	3258.14	399.54	328.09	487.21
Sweden	3803.66	3574.16	3968.14	233.22	207.10	259.30

**Table 2:** Descriptive statistics of the outputs.

Country	Health_LY_65			Life_Expect_M			Life_Expect_F		
	Unit: Absolute Value			Unit: Absolute Value			Unit: Absolute Value		
	Mean	Min	Max	Mean	Min	Max	Mean	Min	Max
Belgium	10.94	10.60	11.10	79.00	78.10	79.80	83.80	83.20	84.30
Bulgaria	9.43	8.80	9.90	71.34	71.10	71.60	78.44	78.00	78.80
Czechia	8.41	8.00	8.90	75.93	75.20	76.40	81.89	81.30	82.20
Denmark	11.63	11.30	12.10	78.94	78.30	79.50	82.89	82.40	83.50
Germany	10.53	6.80	12.20	78.57	78.10	79.00	83.37	83.00	83.70
Estonia	5.87	5.30	6.90	73.43	72.40	74.50	82.33	81.70	83.00
Ireland	12.47	11.50	13.60	79.90	78.90	80.80	83.77	83.10	84.70
Greece	7.64	7.30	7.90	78.89	78.50	79.30	84.04	83.70	84.40
Spain	10.69	9.20	12.40	80.51	80.10	81.10	86.20	85.70	86.70
France	10.46	10.10	11.00	79.49	79.00	79.90	85.77	85.60	86.10
Croatia	5.14	4.60	5.90	74.84	74.40	75.50	81.13	80.50	81.60
Italy	8.94	7.50	10.40	80.81	80.30	81.40	85.41	84.90	85.80
Latvia	4.33	4.10	4.70	69.81	69.10	70.90	79.56	78.90	80.10
Lithuania	5.84	5.30	6.20	69.94	68.50	71.60	80.27	79.60	81.20
Hungary	6.53	5.90	7.20	72.53	72.20	73.10	79.40	79.00	79.70
Netherlands	9.91	9.40	10.50	80.07	79.50	80.60	83.37	83.20	83.70
Austria	7.89	7.40	8.90	79.19	78.60	79.70	83.99	83.70	84.20
Poland	8.20	7.50	8.60	73.69	73.00	74.10	81.70	81.20	82.00
Portugal	7.24	6.10	9.50	78.17	77.60	78.70	84.41	84.00	84.80
Romania	5.93	5.40	6.60	71.60	71.30	71.90	78.97	78.60	79.50
Slovenia	7.87	7.20	8.60	78.11	77.20	78.70	84.11	83.60	84.50
Slovakia	4.16	3.90	4.70	73.59	72.90	74.30	80.60	80.10	81.20
Finland	9.20	8.90	9.50	78.73	78.10	79.30	84.37	83.90	84.80
Sweden	15.51	13.30	16.20	80.69	80.20	81.50	84.20	83.80	84.80

**Table 3:** Summary results of DEA Window efficiency measurement.

Country	CRS				VRS			
	Average	Standard Deviaton	Min	Max	Average	Standard Deviaton	Min	Max
Bulgaria	0.8925	0.0517	0.8365	1.0000	0.9024	0.0463	0.8469	1.0000
Czechia	0.7038	0.0374	0.6367	0.7490	0.7082	0.0351	0.6449	0.7515
Estonia	0.9385	0.0357	0.8840	1.0000	0.9460	0.0317	0.8951	1.0000
Croatia	0.9468	0.0379	0.8812	1.0000	0.9487	0.0375	0.8834	1.0000
Latvia	0.9336	0.0492	0.8616	1.0000	0.9436	0.0416	0.8826	1.0000
Lithuania	0.7533	0.0295	0.7012	0.8015	0.7620	0.0259	0.7151	0.8050
Hungary	0.7704	0.0082	0.7548	0.7824	0.7828	0.0077	0.7699	0.7953
Poland	0.8542	0.0218	0.8309	0.8863	0.8979	0.0458	0.8318	0.9700
Romania	0.9176	0.0730	0.7973	1.0000	0.9208	0.0700	0.8054	1.0000
Slovenia	0.8389	0.0303	0.7823	0.8751	0.8436	0.0303	0.7866	0.8806
Slovakia	0.8281	0.0222	0.7929	0.8564	0.8365	0.0172	0.8114	0.8606
Belgium	0.5789	0.0149	0.5552	0.6029	0.5796	0.0150	0.5559	0.6036
Denmark	0.8779	0.0233	0.8268	0.9024	0.9001	0.0251	0.8471	0.9286
Germany	0.4773	0.0204	0.4463	0.5025	0.4876	0.0254	0.4497	0.5220
Ireland	0.8863	0.0365	0.8541	0.9472	0.8967	0.0437	0.8579	0.9662
Greece	0.9846	0.0077	0.9746	1.0000	0.9914	0.0087	0.9754	1.0000
Spain	0.9861	0.0123	0.9627	1.0000	0.9915	0.0121	0.9655	1.0000
France	0.5653	0.0084	0.5544	0.5750	0.5952	0.0183	0.5780	0.6298
Italy	0.9169	0.0083	0.9046	0.9308	0.9693	0.0347	0.9134	1.0000
Netherlands	0.7523	0.0228	0.7145	0.7831	0.7589	0.0211	0.7245	0.7869
Austria	0.4768	0.0077	0.4686	0.4929	0.4786	0.0080	0.4688	0.4957
Portugal	0.9330	0.0487	0.8499	1.0000	0.9436	0.0462	0.8616	1.0000
Finland	0.7264	0.0646	0.6515	0.8229	0.7382	0.0643	0.6622	0.8353
Sweden	0.9769	0.0278	0.9124	1.0000	0.9824	0.0261	0.9219	1.0000

**Table 4:** (**A**) Results of Malmquist productivity measurement with regard to the previous period. (**B**) Results of Malmquist productivity measurement with regard to the previous period.

**(A)**									
**Country**	**13/14**			**14/15**			**15/16**		
	**MPI**	**EffCh**	**TechCh**	**MPI**	**EffCh**	**TechCh**	**MPI**	**EffCh**	**TechCh**
Austria	0.9652	0.9823	0.9826	0.9796	1.0176	0.9627	1.0160	1.0068	1.0092
Belgium	0.9940	1.0098	0.9844	0.9814	1.0333	0.9498	0.9766	0.9251	1.0557
Bulgaria	0.8838	1.0000	0.8838	0.9675	1.0000	0.9675	1.0016	1.0000	1.0016
Croatia	0.9738	0.9867	0.9869	0.9869	1.0135	0.9738	0.9931	1.0000	0.9931
Czechia	0.9841	1.0320	0.9537	0.9524	0.9833	0.9686	1.0262	1.0103	1.0158
Denmark	1.0697	1.0380	1.0306	1.0352	1.0376	0.9978	0.9914	0.9590	1.0338
Estonia	0.9686	0.9791	0.9893	0.9681	1.0073	0.9610	0.9971	1.0009	0.9962
Finland	1.0215	1.0401	0.9821	0.9963	1.0690	0.9320	1.0496	1.0237	1.0253
France	1.0127	1.0459	0.9682	0.9596	1.0204	0.9404	0.9931	0.9302	1.0676
Germany	0.9844	0.9877	0.9967	1.2477	1.2954	0.9632	0.9999	0.9654	1.0357
Greece	1.0100	1.0000	1.0100	0.9726	1.0000	0.9726	1.0079	1.0000	1.0079
Hungary	0.9898	0.9901	0.9997	0.9780	1.0127	0.9657	1.0113	1.0270	0.9848
Ireland	1.0075	1.0000	1.0075	0.9070	0.9221	0.9837	1.0002	0.9698	1.0314
Italy	1.0173	1.0366	0.9814	0.9791	1.0251	0.9551	1.0409	0.9955	1.0457
Latvia	0.9946	1.0000	0.9946	0.9514	1.0000	0.9514	0.9595	0.9804	0.9787
Lithuania	0.9741	0.9775	0.9966	0.9634	1.0025	0.9610	1.0088	1.0211	0.9879
Netherlands	1.0346	1.0337	1.0009	1.0048	1.0239	0.9813	1.0235	0.9996	1.0238
Poland	1.0045	1.0375	0.9682	0.9636	0.9880	0.9753	1.0150	1.0214	0.9938
Portugal	0.9704	1.0000	0.9704	0.9601	1.0000	0.9601	0.9917	0.9884	1.0034
Romania	0.9848	1.0000	0.9848	0.9586	1.0000	0.9586	0.9409	1.0000	0.9409
Slovakia	1.0387	1.0396	0.9991	0.9759	1.0161	0.9604	1.0372	1.0429	0.9945
Slovenia	1.0106	1.0175	0.9932	0.9486	0.9828	0.9652	1.0137	0.9985	1.0152
Spain	0.9922	1.0000	0.9922	0.9455	1.0000	0.9455	1.0496	1.0000	1.0496
Sweden	1.1126	1.0000	1.1126	1.0062	1.0000	1.0062	1.0350	1.0000	1.0350
GeoMean all countries	0.9991	1.0095	0.9897	0.9813	1.0170	0.9648	1.0072	0.9940	1.0132
GeoMean countries joined before 2004	1.0141	1.0132	1.0009	0.9954	1.0313	0.9652	1.0132	0.9814	1.0325
GeoMean countries joined in and after 2004	0.9818	1.0052	0.9767	0.9649	1.0005	0.9644	1.0000	1.0092	0.9909
**(B)**									
**Country**	**16/17**			**17/18**			**18/19**		
	**MPI**	**EffCh**	**TechCh**	**MPI**	**EffCh**	**TechCh**	**MPI**	**EffCh**	**TechCh**
Austria	1.0020	1.0109	0.9912	0.9945	0.9949	0.9996	0.9962	1.0059	0.9904
Belgium	1.0099	0.9364	1.0784	0.9845	1.0382	0.9483	0.9741	0.9687	1.0055
Bulgaria	0.9025	1.0000	0.9025	1.0321	1.0000	1.0321	0.9760	1.0000	0.9760
Croatia	0.9748	1.0000	0.9748	0.9601	1.0000	0.9601	0.9576	1.0000	0.9576
Czechia	0.9347	0.9512	0.9826	0.9621	0.9821	0.9796	0.9425	0.9258	1.0180
Denmark	0.9880	0.9674	1.0212	0.9853	0.9820	1.0034	0.9954	0.9888	1.0068
Estonia	0.9733	0.9770	0.9963	0.9697	0.9718	0.9978	0.9626	0.9724	0.9899
Finland	1.1328	1.1493	0.9856	0.9296	0.9310	0.9985	1.0407	1.0445	0.9964
France	1.0061	0.9889	1.0174	1.0149	1.0526	0.9642	0.9919	0.9779	1.0143
Germany	0.9685	0.9033	1.0723	0.9704	1.0396	0.9334	0.9981	0.9633	1.0362
Greece	1.0014	1.0000	1.0014	0.9865	1.0000	0.9865	1.0078	1.0000	1.0078
Hungary	1.0121	1.0580	0.9566	1.0003	1.0349	0.9665	0.9786	1.0182	0.9611
Ireland	0.9985	0.9896	1.0090	0.9881	0.9883	0.9997	1.0070	1.0046	1.0024
Italy	0.9882	1.0089	0.9795	0.9957	1.0046	0.9911	0.9786	0.9949	0.9837
Latvia	1.0001	1.0200	0.9804	0.9496	0.9969	0.9525	0.9465	0.9882	0.9579
Lithuania	0.9876	1.0104	0.9775	0.9727	1.0054	0.9675	0.9213	0.9502	0.9696
Netherlands	1.0235	1.0147	1.0087	0.9979	0.9978	1.0001	1.0119	1.0085	1.0034
Poland	0.9675	1.0087	0.9592	0.9982	1.0065	0.9918	0.9673	0.9792	0.9878
Portugal	0.9952	1.0118	0.9837	0.9587	0.9625	0.9961	0.9640	0.9775	0.9863
Romania	0.9107	1.0000	0.9107	0.9042	1.0000	0.9042	0.9131	0.9989	0.9141
Slovakia	1.0237	1.0419	0.9826	0.9975	1.0286	0.9697	0.9568	0.9925	0.9640
Slovenia	0.9871	0.9924	0.9947	0.9858	0.9837	1.0021	0.9663	0.9753	0.9907
Spain	1.0496	1.0000	1.0496	0.9499	1.0000	0.9499	1.0172	1.0000	1.0172
Sweden	1.0259	1.0000	1.0259	1.0148	1.0000	1.0148	1.0367	1.0000	1.0367
GeoMean all countries	0.9934	1.0008	0.9926	0.9789	0.9997	0.9792	0.9790	0.9887	0.9902
GeoMean countries joined before 2004	1.0139	0.9972	1.0168	0.9821	0.9989	0.9832	1.0013	0.9948	1.0066
GeoMean countries joined in and after 2004	0.9696	1.0050	0.9648	0.9751	1.0007	0.9744	0.9533	0.9816	0.9712

## Data Availability

Please contact the corresponding author for a data request.
